# Comparative transcriptomic analyses of normal and malformed flowers in sugar apple (*Annona squamosa* L.) to identify the differential expressed genes between normal and malformed flowers

**DOI:** 10.1186/s12870-017-1135-y

**Published:** 2017-10-23

**Authors:** Kaidong Liu, Haili Li, Weijin Li, Jundi Zhong, Yan Chen, Chenjia Shen, Changchun Yuan

**Affiliations:** 1 Life Science and Technology School, Lingnan Normal University, Zhanjiang, Guangdong 524048 People’s Republic of China; 2 College of Life and Environmental Sciences, Hangzhou Normal University, Hangzhou, 310036 China

**Keywords:** *A. squamosa*, Normal flower, Malformed flower, Transcriptome, Digital gene expression, Phytohormone

## Abstract

**Background:**

Sugar apple (*Annona squamosa* L.), a popular fruit with high medicinal and nutritional properties, is widely cultivated in tropical South Asia and America. The malformed flower is a major cause for a reduction in production of sugar apple. However, little information is available on the differences between normal and malformed flowers of sugar apple.

**Results:**

To gain a comprehensive perspective on the differences between normal and malformed flowers of sugar apple, cDNA libraries from normal and malformation flowers were prepared independently for Illumina sequencing. The data generated a total of 70,189,896 reads that were integrated and assembled into 55,097 unigenes with a mean length of 783 bp. A large number of differentially expressed genes (DEGs) were identified. Among these DEGs, 701 flower development-associated transcript factor encoding genes were included. Furthermore, a large number of flowering- and hormone-related DEGs were also identified, and most of these genes were down-regulated expressed in the malformation flowers. The expression levels of 15 selected genes were validated using quantitative-PCR. The contents of several endogenous hormones were measured. The malformed flowers displayed lower endogenous hormone levels compared to the normal flowers.

**Conclusions:**

The expression data as well as hormone levels in our study will serve as a comprehensive resource for investigating the regulation mechanism involved in floral organ development in sugar apple.

**Electronic supplementary material:**

The online version of this article (10.1186/s12870-017-1135-y) contains supplementary material, which is available to authorized users.

## Background

Sugar apple (*Annona squamosa* L.), a member of the *Annonaceae* family, is an important medicinal plant that is widely distributed in tropical South Asia and America [1]. In addition to its pharmaceutical value, sugar apple is used in juices, jellies and compotes [2]. Due to its medicinal and nutritional properties, sugar apple is a popular tropical fruit in China [3]. The flowering of sugar apple is a complex process that is regulated by various environmental factors, such as improper shoot state, low temperature or short day conditions [4]. Malformed flowers are the major reason for the low percentage of fertile fruit in cultivated sugar apple. However, the available information on the floral organ development-related genes in the genus *Annona* is very scarce.

Flowering is a vital developmental process in the life cycles of plants [5]. The coordinated transition from vegetative growth to reproductive development is required for successful reproduction in higher plants [6]. In the model plant *Arabidopsis thaliana*, the specification of floral organ identities is regulated by a large number of floral genes, most of which encode transcription factors. Based on the phenotypes of *Arabidopsis* mutants, a classical ABC model was developed [7]. According to the model, most of the floral organ identity-related genes can be grouped into three gene classes: A, B and C. In detail, sepals are identified by Class A genes; petals are identified by Class A together with Class B genes; stamens are identified by Class B genes together with Class C genes; and carpels are identified by Class C genes [8]. Recently, the ABC model was extended and modified by genes (Class E) that are essential for the specification of all types of floral organs [9]. The functions of homeotic genes are highly conserved across different species, allowing us to analyze and identify potential genes that are related to flower development in sugar apple.

Various environmental and endogenous signals are associated with an array of biochemical and cellular processes during the formation of floral organs [10]. Among the signals, phytohormones are endogenously occurring compounds involved in the floral transition, flowering and floral organ development [11]. Several classical phytohormones, such as gibberellins (GAs), auxins, cytokinins (CKs), ethylene, abscisic acid (ABA), jasmonates (JAs), salicylic acid (SA) and brassinosteroids (BRs), have been implicated in the flowering-time pathway [11]. GAs are a class of phytohormones that function not only to induce the transition to flowering, but also to control flowering-time [12]. The role of GAs in the floral transition reflects their recognized function as integrators through DELLA-mediated pathways [13]. GAs promote flowering by triggering the expression of a series of floral integrator encoding genes, including *SUPPRESSOR OF OVEREXPRESSION OF CONSTANS 1*, *LEAFY* and *FLOWERING LOCUS T*, (in the inflorescence and floral meristems [14, 15]. Auxins, another classical hormone with a floral-inductive signalling role, were reported as regulators of embryonic and postembryonic development. There is a close correlation between the endogenous auxin content and the flowering time [16]. Several proteins, such as TRANSLOCATED PROMOTER REGION, SUPPRESSOR OF AUXIN RESISTANCE3 and HASTY, are involved in both auxin responses and flowering-time control [17]. CKs, together with auxins, regulate both the division cycle and meristem homeostasis, and may promote the floral transition [18]. In *Arabidopsis*, applications of exogenous CKs accelerate flowering, though the molecular mechanism underlying this is largely unknown [19].

Thus, there is limited data on the molecular basis of floral initiation and differentiation in the genus *Annona*. Without reference genomes, de novo sequencing is an effective approach for the identification of candidate genes in most trees, including melon, litchi and Chinese cherry [20–22]. In our study, two independent cDNA libraries, one of a normal flower and one of a malformed sugar apple flower, were constructed for Illumina RNA sequencing (RNA-seq). The annotation of the transcriptome sequences allowed for the identification and analysis of potential genes related to floral organ development in sugar apple.

## Methods

### Plant material and sampling

In our study, 10-year-old adult trees of *A. squamosa* cv. ‘Bendi’ were cultivated in a 4 × 4 m arrangement with drip irrigation and fertilizer applications as required. The location of the trees was the Ling Nan Normal University field experimental station in Zhanjiang City (Guangdong Province, China). The flowers before open were collected from a normal flower and a malformed flower. All of the flower samples were frozen immediately in liquid nitrogen, and stored at −80 °C for further studies.

### cDNA library preparation and Illumina sequencing for transcriptome analysis

Total RNA was extracted using a TRIzol Kit according to the manufacturer’s protocol (Promega, Beijing, China). Residual DNA contamination was removed by RNase-free DNase I (TaKaRa, Dalian, China). RNA quality was verified by RNase-free agarose gel electrophoresis, and the total RNA concentration was measured using a 2100 Bioanalyzer (Agilent Technologies, Santa Clara, CA, USA) at 260 nm and 280 nm. RNA samples with 260 nm/280 nm ratios between 1.8 and 2.0 were used for subsequent analyses. Each cDNA library was constructed by mixing three independent replicate samples. The library of the normal flower was named ‘NF’, while the library of the malformed flower was named ‘MF’. The two libraries were used for the comparative analysis of transcriptome sequencing using the Illumina HiSeq™ 2500 platform by Gene Denovo Co. (Gene Denovo, Guangzhou, China). Raw reads were generated in a paired-end format, and the NF and MF transcriptome data sets were deposited in the GenBank Short Read Archive under accession number SRA508784.

### De novo assembly and functional annotation of Illumina sequencing

Low-quality reads (with more than 5% unknown bases) and adaptor sequences were filtered and removed, and then the clean reads were assembled using Trinity software to generate unique consensus contigs [23]. All of the contigs were calculated using a sequence clustering software, and the longest sequences were defined as unigenes. The assembled sugar apple unigenes were aligned to several protein databases, such as NCBI Nr protein (http://www.ncbi.nlm.nih.gov), Swiss-Prot protein (http://www.expasy.ch/sprot), Kyoto Encyclopedia of Genes and Genomes (KEGG) pathway (http://www.genome.jp/kegg) and Clusters of Orthologous Groups (COG) (http://www.ncbi.nlm.nih.gov/COG), using the BLASTX algorithm with an E-value <0.00001. For unigene annotation, the Blast2GO program was used to produce the gene ontology (GO) annotation of unigenes, and a BLAST algorithm-based search against the KEGG database (http://www.genome.jp/kegg/) was used to analyze protein products of metabolic processes and related gene functions in cellular processes [24].

### Analysis and mapping of digital gene expression (DGE) tags

To map DGE tags, raw data were filtered to remove the low quality tags (sequences with a number of unknown ‘N’s), empty tags (sequences only containing the adaptors), and tags with only one copy number (sequences from sequencing errors). For annotation, cleaned tags containing CATG and the 21-bp tag sequence were mapped to our transcriptome reference database. Initially, the tags that mapped to multiple genes were filtered out. Then, the remaining tags were treated as unambiguous tags for the gene expression analysis. The number of unambiguous tags of each gene was calculated and then normalized to the number of transcripts per million clean tags. The differentially expressed tags between NF and MF were used for further analysis and mapping.

### Identification of differentially expressed genes (DEGs)

Reads assoicated with each unigene were mapped to the transcriptome using the alignment software Bowtie 0.12.8 (https://sourceforge.net/projects/bowtie-bio/files/bowtie/0.12.8/). To calculate unigene expression, the number of mapped reads for each unigene was counted and normalized into a reads per kb per million reads (RPKM) value. To determine significant differences, a false discovery rate < 0.001 and an absolute value of log_2_ ratio > 1 were set as thresholds. DEGs between the two flower samples were calculated using the edgeR package [25]. GO and KEGG enrichment analyses of the DEGs between two flower samples were performed according to a previous reported method [26].

### Quantitative real-time PCR (qRT-PCR) validation

Total RNA was extracted from the same sample that were used for sequencing. In total, 3 μg RNA of each sample was used for reverse transcription. The cDNA was synthesized using ReverAid First Strand cDNA Synthesis Kit (Thermo Scientific. Shanghai, China). QRT-PCR was performed using the SYBR Premix Ex Taq Kit (TaKaRa, Dalian, China) and an ABI PRISM 7700 DNA Sequence Detection System (Applied Biosystems, Shanghai, China). Three independent cDNA samples from “NF” and “MF” were used for qRT-PCR validation. The primer sequences were designed using Primer Premier 5 software (Premier Biosoft International, Palo Alto, CA, USA). The sugar apple *Actin* gene was used as an internal standard to calculate relative fold-differences based on comparative cycle threshold (2^−ΔΔ*Ct*^) values. Then, ddH_2_O was used as no-template control. The qRT-PCR procedure was as follows: 1 μL of a 1/10 dilution of cDNA in H_2_O was added to 5 μL of 2× SYBR® Green buffer, with 0.1 μM of each primer and H_2_O to a final volume of 10 μL. The reactions were run as follows: 50 °C for 2 min and 95 °C for 10 min, followed by 40 cycles of 95 °C for 30 s, 56 °C for 30 s and 72 °C for 30 s in 96-well optical reaction plates [27]. Each real-time PCR reaction was performed three times.

### Measurements of various hormones

For the exogenous hormone contents analysis, independent samples from normal and malformed sugar apple flowers were harvested, immediately frozen in liquid nitrogen and stored at −80 °C for further extractions. Exogenous indole-3-acetic acid (IAA) contents were determined using a FOCUS GC-DSQII (Thermo Fisher Scientific Inc., Austin, TX, USA) as described previously [28]. Endogenous GA was measured using the nano-LC-ESI-Q-TOF-MS analysis method described previously [29]. In additionally, ABA and zeatin ribosides (ZRs) were detected using a UFLC-MS/MS system described by Kasote et al. [30].

### Statistical analysis

Significant differences between different samples were calculated using a one-way analysis of variance with a Tukey’s test (at a significance level of α = 0.01) in Excel software. All of the expression analyses were performed for three biological replicates. All reported values represent the averages of three replicates, and data are expressed as the mean plus or minus the standard deviation (mean ± SD).

## Results

### Sequencing, de novo assembly and sequence annotation

**Fig. 1:**
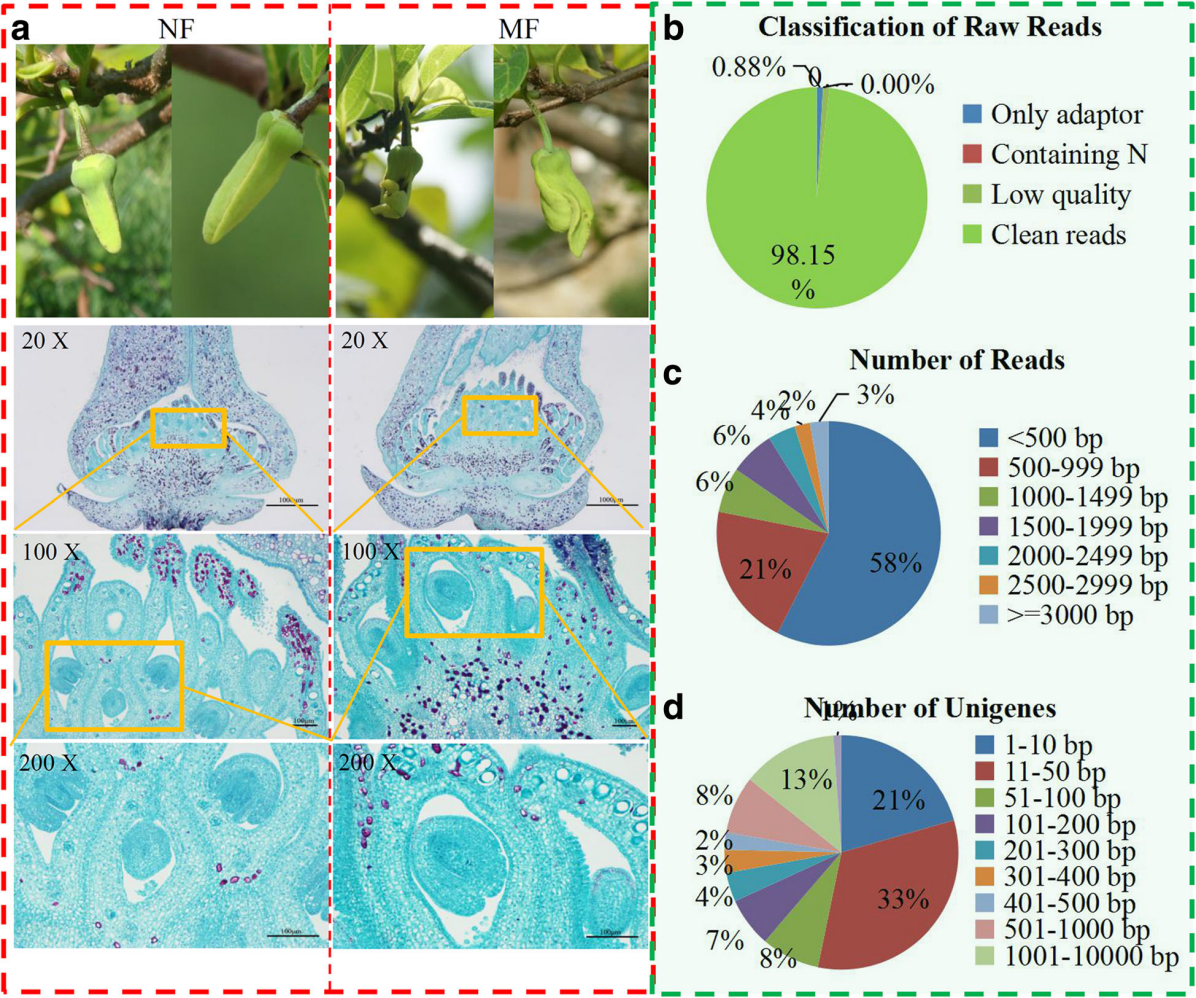
Illumina sequencing of sugar apple flowers. **a** The phenotypes of normal and malformation flowers in sugar apple. **b** Classification of raw reads generated by Illumina sequencing. **c** The length distribution of assembled transcripts in sugar apple. **d** The length distribution of assembled unigenes in sugar apple

To obtain a reference transcriptome for the *A. squamosa* flower, two independent RNA-seq libraries were constructed using RNA samples from normal flowers and malformed flowers, separately. The phenotypic characterizations of normal and malformed flowers is shown in Fig. 1a. In detail, the differentiation and development of petals are irregular in the malformed flowers. Compared with normal flowers at the same stage, the termination of the ovule development occurs before blastophore formation in malformed flowers. Additionally, no female gametes were observed in malformed flowers.

In our study, a large number of raw reads, including 98.15% of clean reads, 0.88% of only adaptors and 0.97% of N-containing reads, were obtained (Fig. 1b). A total of 70,189,896 reads were integrated and assembled into 55,097 unigenes with a mean length of 783 bp. The detailed information of the obtained reads is listed in Additional file 1. The size distributions of clean reads and unigenes in sugar apple are shown in Fig. 1c and d. For clean reads, 3% of the reads were >3000 bp in length and a majority of the reads (58%) were <500 bp in length. Only 1% of the unigenes were >10,000 bp in length, and the majority of unigenes were between 11 bp and 50 bp.

**Fig. 2:**
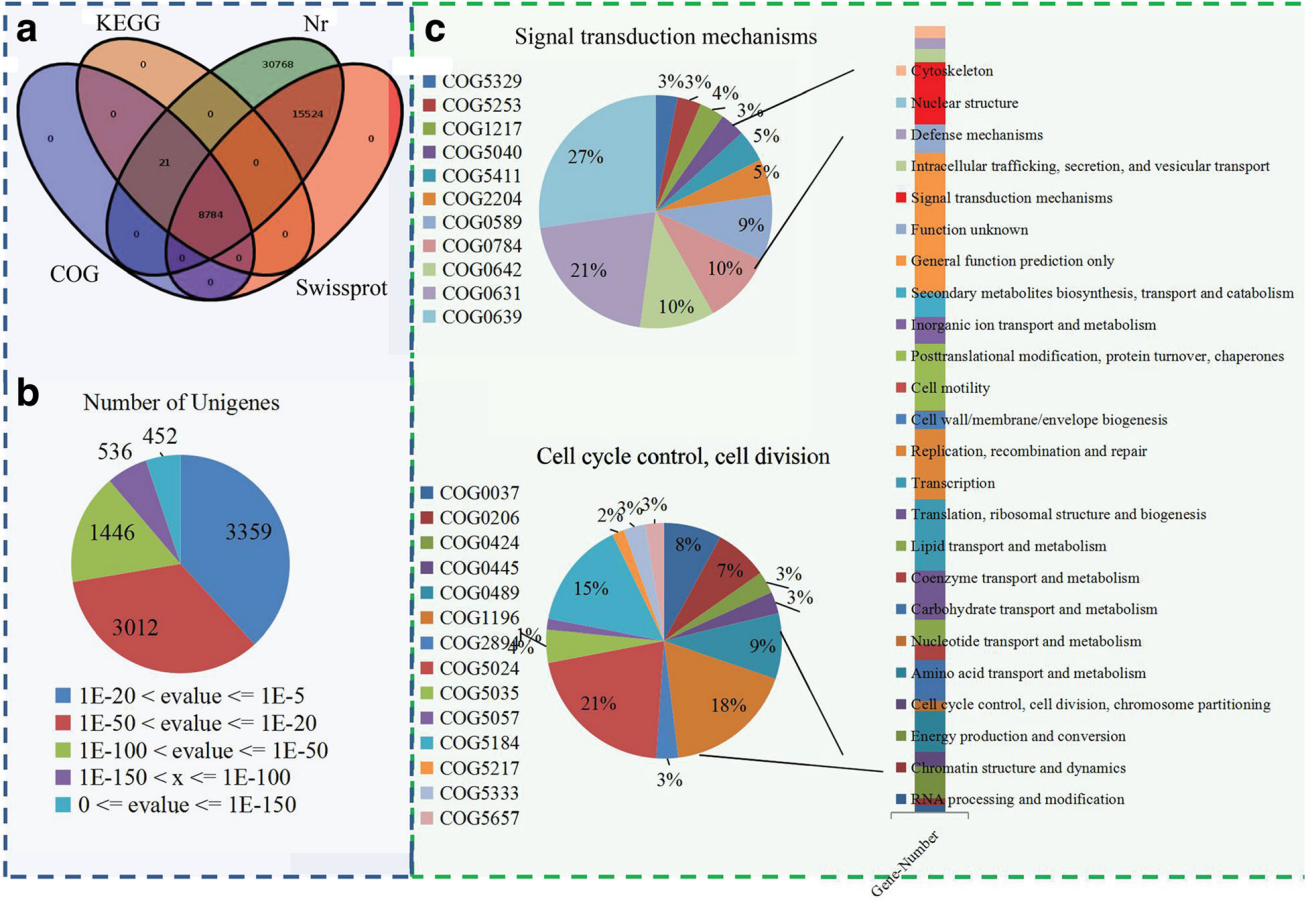
Annotation of assembled sugar apple unigenes. (**a**) The number of unigenes annotated by different databases, including Nr, Swissprot, COG and KEGG, were showed in a Venn diagram. (**b**) Distribution of E value in various databases. (**c**) COG classification of all unigenes of sugar apple. Proportion of each second level COG term belonged to “Signal transduction mechanisms” and “Cell cycle control, cell division”

For the annotation, the unigenes were queried against several databases, including Nr, SwissProt, KEGG and COG, using BLASTX algorithm-based software. In total, 55,097 unigenes could be identified in the Nr database, 24,308 unigenes displayed a significant similarity to known proteins in the SwissProt database, and 8805 unigenes were annotated in both the COG and KEGG databases based on sequence homologies (Fig. 2a). Approximately 8784 unigenes were matched to a homolog in all four databases. The distribution of the E-values of the annotated unigenes is shown in Fig. 2b.

For the COG classification, 13,548 unigenes were grouped into 25 functional classifications. The largest term, “General function prediction only”, contained 2415 unigenes. “Replication, recombination and repair”, “Cell wall/membrane/envelope”, “Posttranslational modification” and “Signal transduction mechanisms” shared a large percentage of unigenes. In addition, only nine unigenes were classified into “Cell motility”, and two unigenes were classified into “Nuclear structure”. Furthermore, two flower development-related COG terms, “signaling transduction mechanisms” and “Cell cycle control, cell division”, were analyzed in detail (Fig. 2c).

### GO and KEGG classifications of unigenes

In sugar apple, most of the unigenes could be assigned to 46 functional terms that belonged to three GO categories, biological process, cellular component and molecular function. For biological process, “metabolic processes” (6128 unigenes) and “cellular processes” (5721 unigenes) were dominant terms; for cellular component, the dominant terms were “cell” (6377 unigenes), “cell part” (6227 unigenes) and “organelle” (6128 unigenes); and for molecular function, a large percentage of unigenes were related to “metabolic process” (6707 unigenes), “catalytic activity” (5942 unigenes) and “binding” (5168 unigenes) (Additional file 2).

Furthermore, all of the unigenes mapped to canonical pathways in the KEGG database. Our data showed that 7191 unigenes from sugar apple were assigned to 124 KEGG pathways (Additional file 3). The most represented KEGG pathway was “metabolic pathways” with 1940 unigenes (ko01100). Additionally, 959 unigenes were associated with the KEGG pathway “biosynthesis of secondary metabolites”, 335 unigenes were mapped to “ribosome”, and 219 unigenes were assigned to “protein processing in endoplasmic reticulum”.

### Screening and classification of DEGs between normal and malformed flowers

**Fig. 3:**
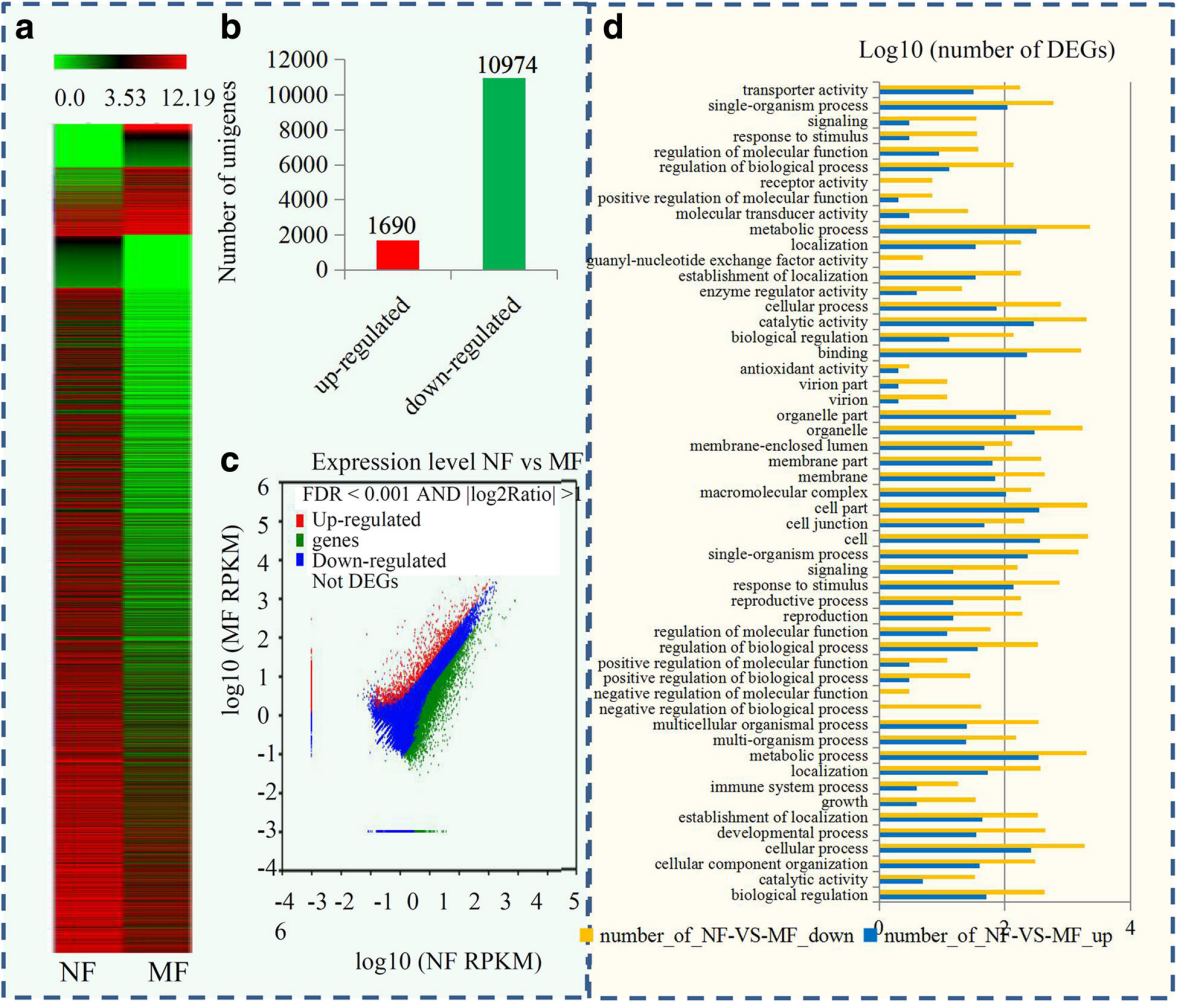
Transcriptional variation normal and malformed flowers in sugar apple. **a** Expression profiles of the differential expressed genes between normal and malformed flowers in sugar apple were showed by a heatmap. **b** The numbers of up-regulated genes and down-regulated genes in malformed flower compared to normal flower. **c** Significance analysis of all DEGs between normal and malformed flowers by a volcanoplot. **d** GO enrichment analysis of DEGs between normal and malformed flowers

To compare the DEGs between normal and malformed flowers, RPKM values were determined to calculate the read density for each unigene. Expression profiles of the DEGs between normal and malformed flowers in sugar apple are shown in a heatmap (Fig. 3a). A total of 12,664 DEGs were identified, including 1690 up-regulated genes and 10,974 down-regulated genes (Fig. 3b). A significance analysis of the DEGs between normal and malformed flowers was visualized using a volcano plot (Fig. 3c). An analysis of the biological functions of these DEGs was performed. Among these DEGs, 2822 unigenes could be annotated from GO-based sequence homologies. For the GO classification, the top five largest GO terms in biological process were “metabolic process”, “cellular process”, “single-organism process”, “response to stimulus” and “localization”; in cellular component, the top five largest GO terms were “cell”, “cell part”, “organelle”, “organelle part” and “macromolecular complex”; and in molecular function, “metabolic process”, “catalytic activity”, “binding”, “single-organism process” and “cellular process” were the five largest GO terms (Fig. 3d).

### Identification of the floral organ development-associated TF-encoding genes and MADS-box genes

In total, 701 *TF* genes were identified as DEGs (Additional file 4). The top five largest differential expressed TF families were: ERF (53 members), bHLH (42 members), MYB-related (38 members), FAR1 (37 members) and NAC (36 members). Moreover, several hormone-related TFs also were identified as DEGs, including 24GRASs and 9 ARFs. In our study, 21 MADS-box genes were revealed from sugar apple flowers (Additional file 5). Among these MADS-box genes, four genes, such as *AGL9*, *SEP1*, *AP1-like*, were identified in MIKC family and 17 genes, such as *AP3-like*, *AGL63* and *PISTILLATA-like*.

### Identification of key flowering- and flower development-related DEGs

A large number of flowering- and flower development-related genes play important roles in the entire flower developmental cycle [31]. In total, 18 DEGs in sugar apple showed homology to known flowering- and flower development-related genes in the NCBI and UniProt databases (Additional file 6). For example, two flowering promoting factor genes (Unigene0041725 and Unigene0054870) showed higher expression levels in the malformed flowers than in the normal flowers. Additionally, four early flowering protein genes (Unigene0023978, Unigene0023976, Unigene0033968 and Unigene0008711), one flowering locus T gene (Unigene0000043), one flowering time control protein FY-like gene (Unigene0025538), three flowering time control protein FPA-like genes (Unigene0005475, Unigene0019814 and Unigene0005474), and one flowering time control protein FCA-like gene (Unigene0024514), were also identified as DEGs.

Based on their GO terms (GO:0009908), 41 DEGs were identified as flower development-related genes (Additional file 7). Interestingly, two genes, a probable histone H2A variant 3-like gene (Unigene0017118) and ODORANT1-like gene (Unigene0047135), were largely induced (> 5 fold) in the malformed flowers. Most of other identified flower development-related DEGs were reduced in the malformed flowers.

### Identification of hormone-related DEGs

**Fig. 4:**
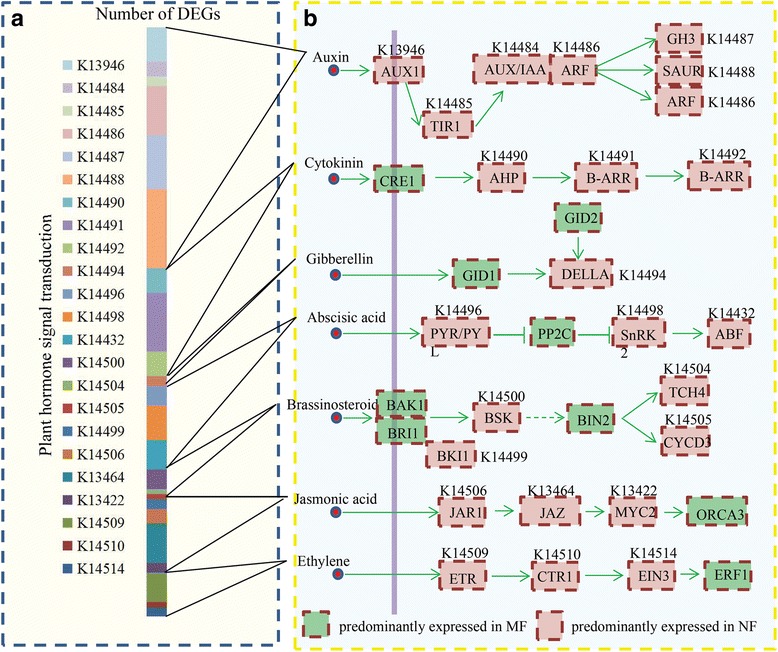
Identification and differential analysis of the hormonal network between normal and malformed flowers in sugar apple. **a** The number of the DEGs encoding the key components involved in various hormonal signaling pathways. **b** Overview of various hormonal signaling network in sugar apple. Red indicated the normal flower predominantly expressed genes and green indicated the malformed flower predominantly expressed genes

In our study, a large number of hormone-related DEGs were identified between normal and malformed flowers. The KEGG analysis assigned most of the DEGs to key components involved in various hormonal signaling pathways (Fig. 4a). Interestingly, a large number of hormone-related DEGs belonged to auxin and CK signaling pathways. An overview of the various hormonal signaling networks in sugar apple is shown in Fig. 4b. Most hormone-related DEGs were predominantly expressed in normal flowers.

**Fig. 5:**
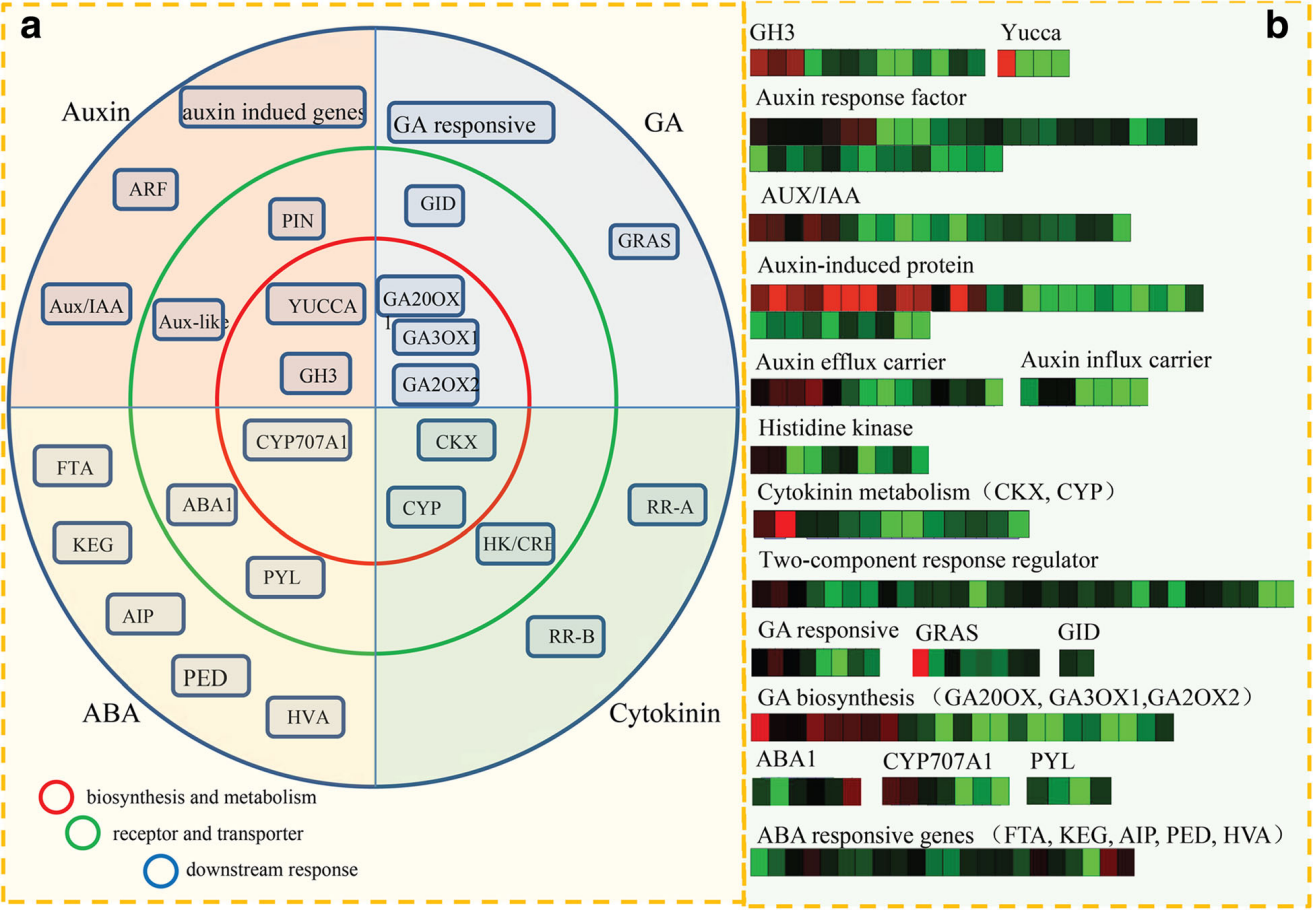
Transcript abundance changes of hormone-related genes. **a** The detailed information on genes involved different hormone signaling pathway. The key components in red cycle indicated genes related to hormone biosynthesis and metabolism; the key components in green cycle indicated genes encoding receptor and transporter; and the key components in blue cycle indicated genes related to downstream response. **b** Expression changes of the genes associated with different hormones, including auxin, ABA, GA and cytokinin. Red indicates up-regulated genes and blue indicates down-regulated genes

Firstly, DEGs related to auxin were analyzed. For auxin biosynthesis and metabolism, 5 *YUCCA* genes and 13 *GH3* genes were identified as DEGs; for auxin transport, 14 efflux carrier-encoding genes and seven influx carrier-encoding genes were identified as DEGs. For the downstream response, 35 auxin-induced genes, 3 auxin-repressed genes, 39 auxin response factor-encoding genes, and 21 auxin/IAA-encoding genes were identified as DEGs. Secondly, DEGs associated with ABA were identified. For ABA biosynthesis, seven *CYP707A1* genes were obtained. For ABA receptors and transporters, seven ABA1-encoding genes and four PYL-encoding genes were identified. For ABA downstream responses, 21 DEGs were annotated as *FTA*, *KEG*, *AIP*, *PED* and *HVA* genes. Then, DEGs related to GA were analyzed. For GA biosynthesis, 23 DEGs encoding GA20OX1, GA3OX1 and GA2OX2 were identified, and 3 DEGs were annotated as GA receptors. For GA downstream responses, eight DEGs were identified as GA responsive genes and eight DEGs were identified as GRAS. Furthermore, 53 DEGs were mapped to the CK pathway. In total, 13 DEGs were associated with CK biosynthesis and metabolism (such as *CYP* and *CKX*), 10 DEGs were identified as HK/CRE-encoding genes, and 31 CK downstream responsive genes were identified (Fig. 5a).

Based on the RPKM values, the expression levels of the hormone-related unigenes were analyzed and shown in Fig. 5b. The expression levels of most hormone-related unigenes in the normal flowers were greater than those in the malformed flowers.

### Validation of the expression of several key hormone-related genes

**Fig. 6:**
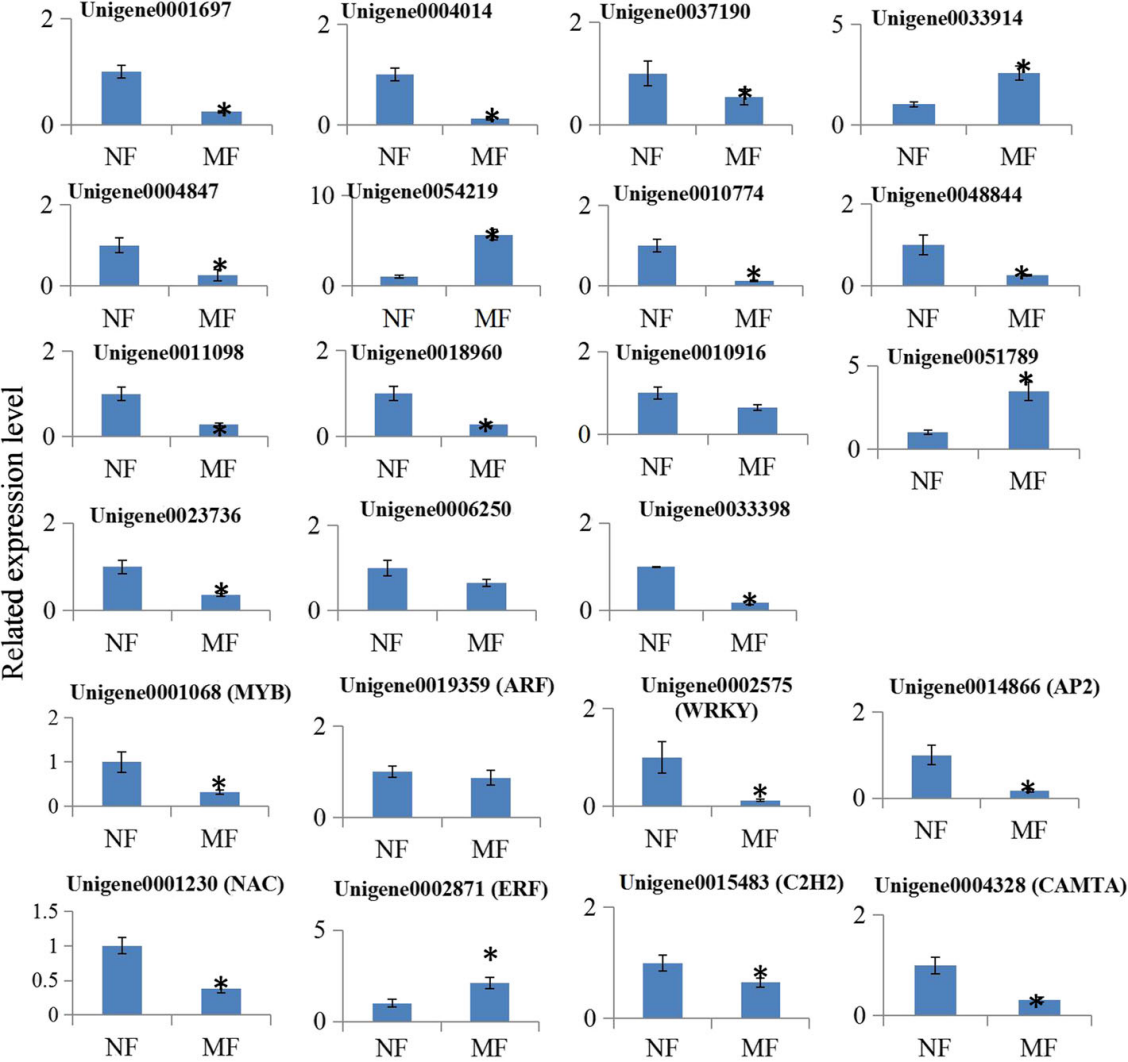
Real-time quantitative PCR validation of several selected hormone-related genes. Total RNA was extracted from normal and malformation flowers. The histogram shows the relative expression level of these genes with respect to the ACTIN in hickory. The specific identities of the genes: Unigene0001697 (auxin-induced protein 5NG4-like), Unigene0004014 (auxin response factor 5-like), Unigene0037190 (IAA29-like), Unigene0033914 (PIN5), Unigene0004847 (gibberellin-regulated protein 9), Unigene0054219 (GRAS family transcription factor), Unigene0010774 (gibberellin 2-oxidase 2), Unigene0048844 (gibberellin 3-beta-hydroxylase), Unigene0011098 (APRR2), Unigene0018960 (PRR95), Unigene0010916 (cytokinin dehydrogenase 3-like), Unigene0051789 (3-ketoacyl-CoA thiolase 2), Unigene0023736 (zeaxanthin epoxidase), Unigene0006250 (ABA 8′-hydroxylase), Unigene0033398 (PYR1-like). The data were analyzed by three independent repeats, and standard deviations were shown with error bars. Significant differences in expression level were indicated by “*”

To verify the differential expression levels of some key hormone-related genes identified by RNA-seq, a qRT-PCR assay with independent samples collected from normal and malformed flowers was performed. In total, 15 key flower hormone-related genes and eight floral organ development-associated TF-encoding genes were randomly selected to validate the RNA-seq data. The expression levels of these selected genes were basically consistent with RNA-seq results (Fig. 6). Furthermore, the expression of these selected genes were analyzed with independent samples collected from the flowers at different developmental stages [32]. Expression profiles of these selected genes during the flower development process were showed in Additional files 8 and 9 **.** The primer sequences are listed in Additional file 10.

### Endogenous hormone measurements

**Fig. 7:**
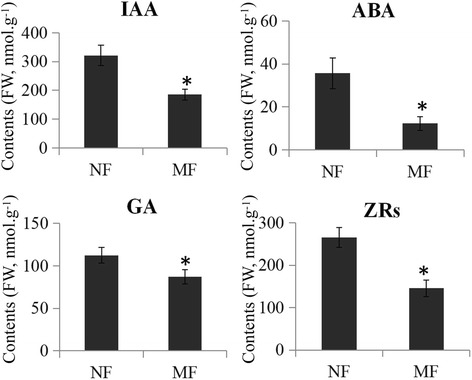
Endogenous hormone measurements. The contents of endogenous hormones, including IAA, ABA, GA and ZRs, between normal and malformation flowers were measured. Three independent samples collected from different flowers were used for endogenous hormone measurements. Significant differences in contents were indicated by “*”

To examine the changes in endogenous hormones between normal and malformed flowers, the IAA, ABA, GA and ZRs contents were measured. Three independent samples collected from different flowers were used for endogenous hormone measurements. The IAA, ABA, GA and ZRs contents were reduced in malformed flowers (Fig. 7). In detail, compared with normal flowers, the IAA contents decreased from 321.23 to 185.25 nmol·g^−1^, the ABA contents decreased from 35.63 to 12.32 nmol·g^−1^, the GA contents decreased from 112.36 to 87.23 nmol·g^−1^, and the ZRs contents decreased from 265.32 to 145.68 nmol·g^−1^ in malformed flowers.

## Discussion

Sugar apple is a popular fruit tree with a high commercial value in subtropical and tropical areas [1]. Without a public genome database, detailed information on the genes involved in their growth and development is unavailable for sugar apple studies. A DEG analysis is a good tool for studying the temporal regulation of gene expression [33]. Our previous work constructed independent cDNA libraries of four different flower stages in *A. squamosa* for Illumina RNA-seq. A large number of flower developmental stage-related genes have been identified [32]. In the present study, transcriptome data from normal and malformed flowers at the same developmental stage (the mature flowers with partially opened petals) were used to uncover the differences between normal and malformed flowers and provide a more adequate resource to study sugar apple.

In model plants, various TF families, such as MYB, MADS-box, NAC, ARF and bHLH, have been reported to be involved in floral development [34–38]. Therefore, the differential expression levels of TF genes between normal and malformed flowers were analyzed in our study. Our previous study identified a large number of flower development-related TF families, including bHLH, NAC, B3, MYB-related and bZIP. MYB factors promote petal and stamen growth in *Arabidopsis*. For example, MYB21 fed back negatively on the expression of JA biosynthesis-related genes to control flower development by decreasing the JA level [39]. Our previous study identified a large MYB-related TFs, suggesting an important role for MYB factors in the floral organ development of sugar apple [32]. In the present study, the MYB family contained the largest number of differentially expressed TFs (59 members). Additionally, bHLH is another large TF family that regulates various flower developmentally related processes [40]. In sugar apple, the bHLH family had the second largest number of differentially expressed TFs (35 members) between normal and malformed flowers. Our previous study showed that bHLH is the largest TF family involved in flower development of sugar apple, indicating a involvement of MYB and bHLH families in the differences between normal and malformed flowers.

The *Arabidopsis* TFs *ARF6* and *ARF8* are expressed in multiple flower tissues, such as sepals, petals and stamen filaments [41]. Moreover, these two genes function in different organs to promote the transition from closed buds to mature fertile flowers [42]. *ARF1* and *ARF2* regulate senescence and floral organ abscission in *Arabidopsis* [43]. In sugar apple, nine ARFs were identified as differentially expressed TFs between normal and malformed flowers, indicating that ARF-mediated auxin signaling participated in flower development.

It has been reported that morphogenesis of floral organs was under control of several MADS-box genes. Loss-of-function of any essential MADS-box genes may result in homeotic conversion of floral organs [44]. For example, four orchid *AGL9-like* MADS-box genes play roles in floral transition and formation in *Arabidopsis* [45]. In the present study, four MIKC-type MADS-box genes were identified, including an *AGL9* gene, which may involved in floral transition of sugar apple. Besides, SEPALLATA subfamily MADS-box protein was reported to positively control spikelet meristem identify in rice [46]. A SEPALLATA 1-like protein encoding gene also was identified in sugar apple. The data provides a more comprehensive information of the floral organ development process in sugar apple.

Based on the DEGs analysis, a large number of auxin-related genes, including *ARF*s, *Aux/IAA*s, and *GH*s, were identified as DEGs. The phytohormone auxin is involved in regulating many aspects of plant growth and development [47, 48]. A classical role of auxin in the formation of flowers at the periphery of the reproductive shoot apex was revealed by genetic evidence [49]. For example, mutations in *PIN1* or *PID*, auxin efflux carrier-encoding genes, lack flowers [50]. At the early stage, the development of female gametophytes is controlled by the *AtPIN1-*mediated auxin flux [51]. In our study, the identified auxin influx carrier-encoding genes and most of the identified auxin efflux carrier-encoding genes were down-regulated in the malformed flowers. In *Arabidopsis*, *ARF4* is associated with flower patterning [52]. Another *ARF* gene, *AtARF3*, integrates the functions of *AGAMOUS* and *APETALA2* in floral meristem determinacy [53, 54]. Here, the expression of most of the identified *ARFs* and *Aux/IAAs* was also reduced in the malformed flowers. Thus, auxin and auxin transport may be required for floral meristem determinacy and flower patterning in sugar apple.

CK plays pivotal roles in many aspects of plant development, including the formation of male and female functions [55]. In *Arabidopsis*, the floral homeotic gene *APETALA1* directly reduces the biosynthesis of endogenous CK by promoting the expression of the CK degradation gene *CYTOKININ OXIDASE/DEHYDROGENASE3* and inhibiting the expression of the CK biosynthetic gene *LONELY GUY1* [56]. In our study, a number of CK biosynthesis-related genes, including 3 *CYPs* and 10 *CKXs*, were identified as DEGs in sugar apple. Decreases in the biosynthesis of CK may cause the malformed flower phenotype.

In additional, GA and ABA play important roles in flower development. For example, effects of exogenous GA_3_ applications on grape flowers have been analyzed. An RNA-seq transcriptome analysis suggested that the morphology of grape inflorescences may be controlled by the biosynthesis and signaling of GA_3_ [57]. In *Arabidopsis*, a GA-deficient mutant, *ga1–3*, displayed the retarded growth of four whorls in the floral organs, and its flower phenotypes could be rescued by the application of exogenous GA [58]. GA-INSENSITIVE DWARF1 (GID1), a soluble protein with a high similarity to hormone-sensitive lipases, was first cloned in rice as a GA receptor [59]. In our study, two GID-encoding genes were identified as DEGs, suggesting an involvement of GA signaling in sugar apple’s flower development. For GA biosynthesis, the *GA2ox* family genes were identified in various plant species [60]. Based on our transcriptome data, some GA3OX1 family genes, such as Unigene0016441, were largely reduced in malformed flowers, while several GA2OX2 family genes, such as Unigene0010774 and Unigene0004102, were induced in malformed flowers. This suggested that a diversity of regulatory mechanisms are involved in GA biosynthesis during flower development. A number of transcriptomes revealed that there is a close relationship between ABA signaling and flower development in various plant species, such as *Gerbera hybrida* [61]. Our data confirmed that ABA-related genes also showed significant expression changes between normal and malformed flowers.

Furthermore, malformed flowers displayed lower endogenous hormone levels compared with the normal flowers. This was in agreement with the decreases in the expression levels of hormone-related genes in malformed flowers. In *Arabidopsis*, increased auxin levels attribute the initiation of flower formation in the shoot apex [62]. Addition to auxin, cytokinin accumulation also causes alteration of flower development in *Arabidopsis* [63]. In sugar apple, low auxin and cytokinin levels may affect downstream processes of flower development and lead to malformed flowers. ABA alters the number of carpels in *Arabidopsis* [64]. Compared with normal flowers, lower ABA level may affect the formation of carpels in malformed flowers. Recent publications have established that GA is involved in stamen development. For example, mutations in GA receptors affect the elongation of stamens [65]. GA concentration may play a role in stamen development in sugar apple.

In our previous work, genes associated with floral transition and flower development were identified in sugar apple [32]. In the present study, most selected DEGs showed significantly expression changes during the flower development process, suggesting a causal relationship to the defects associated with the malformed flowers.

## Conclusions

In summary, two independent cDNA libraries from normal and malformed flowers of sugar apple were separately constructed and sequenced. A large number of DEGs were identified between normal and malformed flowers. The expression changes of hormone-related unigenes were analyzed and validated using a qRT-PCR analysis. The expression analysis, together with the hormone contents, provides a comprehensive resource for investigating the differential expressed genes between normal and malformed flowers.

## Additional files

Additional file 1: The detailed information of the obtained reads. (XLSX 8 kb)

Additional file 2: GO classifications of unigenes. (XLSX 10 kb)

Additional file 3: KEGG classifications of unigenes. (XLSX 13 kb)

Additional file 4: The information of 701 TF genes were identified as DEGs. (XLSX 21 kb)

Additional file 5: The information of 21 MADS-box genes were revealed from sugar apple flowers. (XLSX 15 kb)

Additional file 6: Identification of key flowering- and flower development-related DEGs. (XLSX 12 kb)

Additional file 7: The gene list of GO:0009908. (XLSX 10 kb)

Additional file 8: Expression profiles of 15 key flower hormone-related genes during the flower development process. (DOCX 658 kb)

Additional file 9: Expression profiles of eight floral organ development-associated TF-encoding genes during the flower development process. (DOCX 560 kb)

Additional file 10: The primer sequences. (XLSX 10 kb)

## Abbreviations

ABA: abscisic acid
BRs: brassinosteroids
CKs: cytokinins
COG: Clusters of Orthologous Groups
DEGs: differentially expressed genes
DGE: digital gene expression
GAs: gibberellins
GO: gene ontology
IAA: indole-3-acetic acid
JAs: jasmonates
KEGG: Kyoto Encyclopedia of Genes and Genomes
qRT-PCR: quantitative real-time PCR
RNA-seq: RNA sequencing
RPKM: reads per kb per million reads
SA: salicylic acid
ZRs: zeatin ribosides
