# Supplementary material for: Comparative transcriptomic analyses of normal and malformed flowers in sugar apple (Annona squamosa L.) to identify the differential expressed genes between normal and malformed flowers
Source: BMC Plant Biol. 2017 Oct 23;17:170. doi: 10.1186/s12870-017-1135-y (PMC5653983; doi:10.1186/s12870-017-1135-y)
Supplement: Supplementary file 8 — Expression profiles of 15 key flower hormone-related genes during the flower development process. (DOCX 658 kb) [file 12870_2017_1135_MOESM8_ESM.docx]

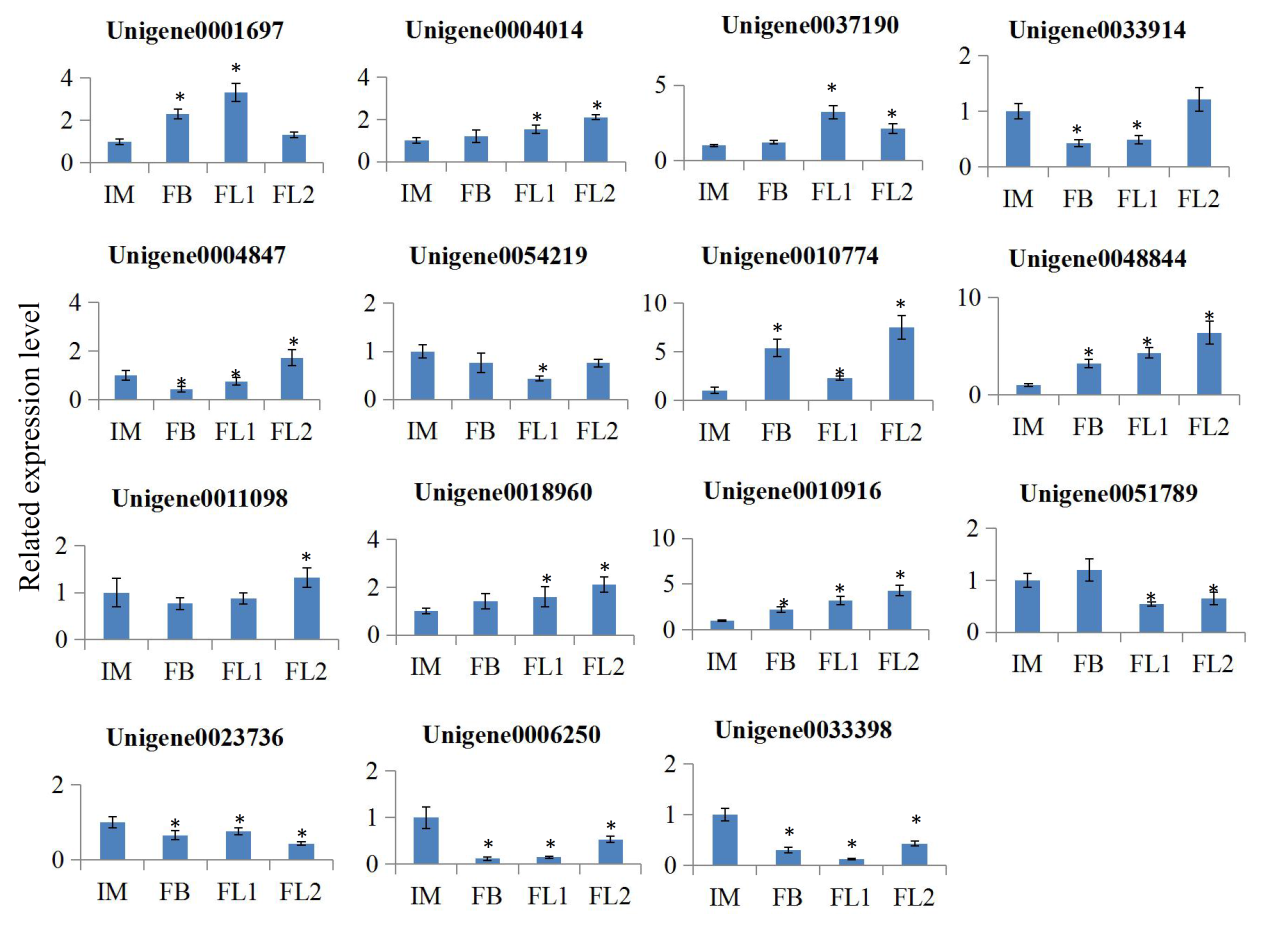


Figure S1 Expression profiles of 15 key flower hormone-related genes during the flower development process. The expression level of these genes in IM is set as control, and the histogram shows the relative expression level of these genes among various samples. The specific identities of the genes: Unigene0001697 (auxin-induced protein 5NG4-like), Unigene0004014 (auxin response factor 5-like), Unigene0037190 (IAA29-like), Unigene0033914 (PIN5), Unigene0004847 (gibberellin-regulated protein 9), Unigene0054219 (GRAS family transcription factor), Unigene0010774 (gibberellin 2-oxidase 2), Unigene0048844 (gibberellin 3-beta-hydroxylase), Unigene0011098 (APRR2), Unigene0018960 (PRR95), Unigene0010916 (cytokinin dehydrogenase 3-like), Unigene0051789 (3-ketoacyl-CoA thiolase 2), Unigene0023736 (zeaxanthin epoxidase), Unigene0006250 (ABA 8'-hydroxylase), Unigene0033398 (PYR1-like). The inflorescent meristem (IM), the flower buds (FB), and two stages of flowers (FL1 and FL2) were used. The two flower stages were the mature flowers with partially opened petals (FL1) and mature flowers with opened and faded petals (FL2). The data were analyzed by three independent repeats, and standard deviations were shown with error bars. Signiﬁcant differences in expression level were indicated by “*”.
