# Supplementary material for: Comparative transcriptomic analyses of normal and malformed flowers in sugar apple (Annona squamosa L.) to identify the differential expressed genes between normal and malformed flowers
Source: BMC Plant Biol. 2017 Oct 23;17:170. doi: 10.1186/s12870-017-1135-y (PMC5653983; doi:10.1186/s12870-017-1135-y)
Supplement: Supplementary file 9 — Expression profiles of eight floral organ development-associated TF-encoding genes during the flower development process. (DOCX 560 kb) [file 12870_2017_1135_MOESM9_ESM.docx]

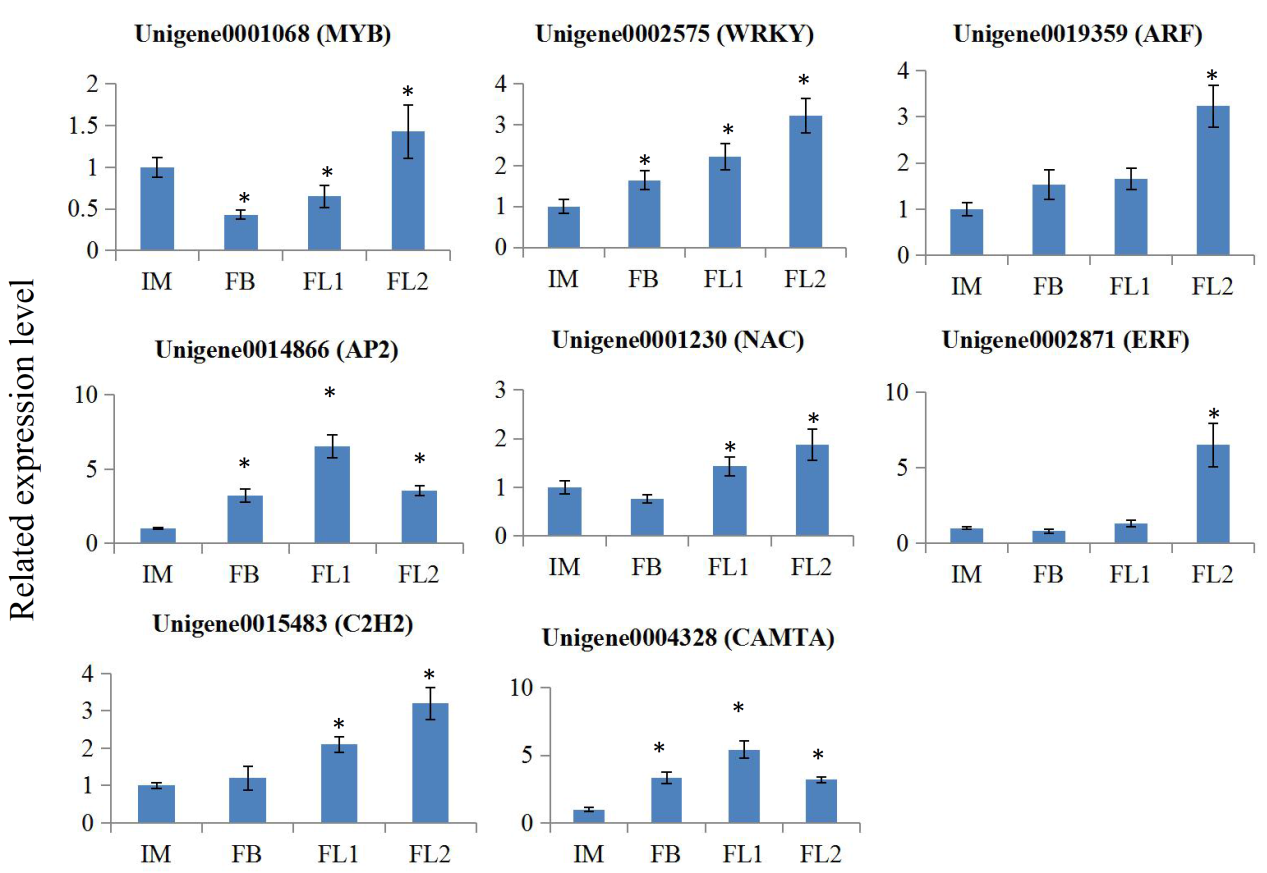


Figure S2 Expression profiles of eight floral organ development-associated TF-encoding genes during the flower development process. The expression level of these genes in IM is set as control, and the histogram shows the relative expression level of these genes among various samples. The inflorescent meristem (IM), the flower buds (FB), and two stages of flowers (FL1 and FL2) were used. The two flower stages were the mature flowers with partially opened petals (FL1) and mature flowers with opened and faded petals (FL2). The data were analyzed by three independent repeats, and standard deviations were shown with error bars. Signiﬁcant differences in expression level were indicated by “*”.
